# Brain responses to painful electrical stimuli and cognitive tasks interact in the precuneus, posterior cingulate cortex, and inferior parietal cortex and do not vary across the menstrual cycle

**DOI:** 10.1002/brb3.2593

**Published:** 2022-05-05

**Authors:** Dieuwke S. Veldhuijzen, Timothy J. Meeker, Deborah Bauer, Michael L. Keaser, Rao P. Gullapalli, Joel D. Greenspan

**Affiliations:** ^1^ Institute of Psychology Health, Medical and Neuropsychology Unit Leiden University Leiden the Netherlands; ^2^ Leiden Institute for Brain and Cognition Leiden the Netherlands; ^3^ Department of Neurosurgery Johns Hopkins University Baltimore Maryland; ^4^ Department of Neural and Pain Sciences University of Maryland School of Dentistry Baltimore Maryland; ^5^ Center to Advance Chronic Pain Research University of Maryland Baltimore Baltimore Maryland; ^6^ Department of Diagnostic Radiology and Nuclear Imaging University of Maryland School of Medicine Baltimore Maryland

**Keywords:** cognitive performance, electrical pain, gonadal hormones, menstrual cycle, MSIT

## Abstract

**Introduction:**

Bidirectional effects between cognition and pain have been extensively reported. Although brain regions involved in cognitive and pain processing seem to partly overlap, it is unknown what specific brain regions are involved in the interaction between pain and cognition. Furthermore, the role of gonadal hormones on these interacting effects has not been examined. This study investigated brain activation patterns of the interaction between pain and cognition over different phases of the naturally occurring menstrual cycle.

**Methods:**

Fifteen healthy normally cycling females were examined over the course of 4 different cycle phases. Sensory stimulation was applied using electrical pulses and cognitive performance was assessed using the Multi‐Source Interference Task. Brain imaging consisted of functional magnetic resonance imaging using a repeated measures ANOVA group analysis approach.

**Results:**

Sensory stimulation was found to interact with task performance in the left precuneus, left posterior cingulate cortex and right inferior parietal lobule. No effects of cycle phase were observed to interact with main effects of stimulation, task or interaction effects between task performance and sensory stimulation.

**Conclusion:**

Potential neural correlates of shared resources between pain and cognition were demonstrated providing further insights into the potential mechanisms behind cognitive performance difficulties in pain patients and opening avenues for new treatment options including targeting specific cognitive factors in pain treatment such as cognitive interference.

## Graphical abstract

Neural correlates of shared resources between pain and cognition were demonstrated in the precuneus, cingulate gyrus, and inferior parietal lobule. There were no significant effects of interactions of menstrual cycle phase.

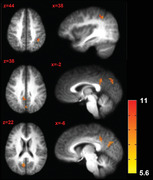

## 1 INTRODUCTION

Bidirectional effects between cognition and pain have been extensively investigated in the behavioral realm (Eccleston, 1995; Seminowicz & Davis, 2007a; Seminowicz et al., 2004; Van Ryckeghem et al., 2013; Vogt et al., 1996). Pain may influence cognitive task performance, whereas cognitive task performance may alternatively influence pain perception (Moore et al., 2017; Moore et al., 2012; Van Ryckeghem et al., 2013; Veldhuijzen et al., 2006). Moreover, cognitive impairments are frequently observed in pain patients (Oosterman et al., 2012). Several mechanisms have been proposed to underlie these interactions between pain and cognition. The limited resource theory for example postulates that cognition and pain compete for finite shared information processing resources (Eccleston & Crombez, 1999; Handy, 2000; Legrain et al., 2009). As such, multimodal input requires prioritization, which could lead to an orienting response to inherently salient nociceptive input, or to a distraction away from pain (Roelofs et al., 2004). It has been further hypothesized that pain processing may depend on cognitive inhibitory processes (Moore et al., 2012; Oosterman et al., 2012). In support of these different accounts, brain regions involved in cognitive and pain processing have been reported to overlap to some extent (Legrain et al., 2009; Seminowicz & Davis, 2007a; Seminowicz et al., 2004). However, the study findings are mixed as some reports did not find an explicit interaction of cognitive task performance and pain in the brain whereas other studies did present such interaction however used indirect statistical methods (e.g., partial least square analysis, PLS) (Seminowicz & Davis, 2007a, 2007b). It therefore remains currently unknown which brain regions are specifically involved in the interaction between pain and cognition.

Gonadal hormones, including estrogen and progesterone, may potentially influence these interactions between cognition and pain. It has been reported that pain is differentially experienced across the menstrual cycle, and we and others demonstrated that brain responses to painful stimuli differ over the cycle (Choi et al., 2006; Iacovides et al., 2015; Veldhuijzen et al., 2013). Furthermore, evidence suggests cognitive performance might is affected by gonadal hormones (Beltz & Moser, 2020). The role of gonadal hormones on the interaction between cognition and pain has however not yet been examined.

This study aimed to investigate the interaction between pain and cognition using functional magnetic resonance imaging. To this aim, we used a previously validated paradigm for studying interactions of pain and cognitive load, the Multi‐Source Interference Task (Bush & Shin, 2006). In addition, the role of gonadal hormones in these processes was explored by examining these interactions over the different phases of the naturally occurring menstrual cycle representative of different gonadal hormone states.

## 2 METHODS

### 2.1 Participants and menstrual cycle timing

Healthy, normally cycling females were eligible to participate; full eligibility criteria were published previously (Meeker et al., 2020; Veldhuijzen et al., 2013). The current study presents new data on cycle effects on painful *electrical* stimuli *combined* with a cognitive task. In brief, blood hormone assessments ensured participants attended experimental sessions during appropriate cycle phases. Sessions took place during the menstrual (2–4 days after menses onset: low estrogen and low progesterone), midfollicular (6–8 days after menses onset: low estrogen and low progesterone), ovulatory (within 1 day of the first positive ovulation test: high estrogen and low progesterone), and midluteal phases (1 week after ovulation: high estrogen and high progesterone). The University of Maryland, Baltimore Institutional Review Board for the Protection of Human Participants approved the study. All participants provided written, informed consent.

### 2.2 Sensory testing

We delivered 20 Hz electrical stimuli to the left foot dorsum with 2 by 2‐inch electrodes passing a symmetrical biphasic pulse with a pulse width of 200 μs. Sensory detection and pain thresholds were determined. Two stimulus intensities were selected: a nonpainful (Tingle) and a moderately painful (Pain) stimulus, which was rated 60 on a 0–100 visual analog scale (VAS).

### 2.3 Multi‐Source Interference Task

The MSIT consisted of three difficulty levels: a simple motor tapping task (simple motor [SM]), a neutral (N) task with congruent stimuli, and a high cognitive demand task with incongruent stimuli (interference [I]) (Bush & Shin, 2006; Seminowicz & Davis, 2007a). In the simple motor tapping task, an asterisk moved sequentially from left to right. In the other two tasks, three digits were presented, using either a 1, 2, or 3, where the target number needed to be identified. In the neutral task, a target number appeared between two other numbers in its congruent position (e.g., 100: the correct response is to push first button). In the interference task, the target number was incongruent to its position (e.g., 221: the correct response is to push first button). Participants used their right hands to press an MRI‐compatible button box within 1.5 s.

Electrical stimulus levels or a baseline, no stimulation condition, were paired with all MSIT levels resulting in nine stimulus‐task pairings. We presented each stimulus‐task pair three times during each of two fMRI scans in counter‐balanced orders. We counter‐balanced these conditions in a hierarchical block design (block 1: stimulus condition, block 2: task condition). We presented each condition for 14 s and the participant performed the task nine times during each block. After each scan, participants provided pain intensity and unpleasantness ratings on 0–100 VAS scales for the tingle and pain stimuli.

### 2.4 fMRI

We recorded fMRI on a 3‐T Tim Trio scanner (Siemens Medical Solutions, Malvern, PA) with a 12‐channel head coil with parallel imaging. A gradient echo single‐shot echo‐planar‐imaging sequence provided a 3.6 × 3.6 mm resolution over a 23‐cm field of view (FOV). We accomplished T2*‐weighting with an echo time (TE) of 30 ms and flip angle (FA) of 90°. We covered the whole brain with a repetition time (TR) of 2000 ms acquiring 24 slices of 6 mm thickness interleaved without a slice gap. During each session, two scans of 199 volumes of 6 min 38 s duration were recorded. To allow for anatomical reference, we acquired a 3‐dimensional T1 magnetization‐prepared rapid gradient echo (MPRAGE) volumetric scan with 3.44 ms TE, 2250 ms TR, 900 ms tissue inversion (TI) time, FA 9°, 96 slices, slice thickness 1.5 mm, and 0.9 × 0.9 mm in‐plane resolution over a 23‐cm FOV.

### 2.5 Data analysis

For MRI data analysis, Analysis of Functional NeuroImages (AFNI: http://afni.nimh.nih.gov) was used. The first four volumes of both MSIT functional scans were removed to allow for signal equilibration. Preprocessing involved slice timing correction, coregistration, spatially alignment to the first volume of the first MSIT scan, spatial normalization to Talairach space, spatial smoothing with a 5 mm full‐width, half‐maximum Gaussian blur, and applying AFNI's 3dDespike. The processed functional data were scaled on a voxel‐wise basis to a mean of 100. A general linear model (GLM) modeled BOLD responses assuming a standard boxcar regressor convolved with the hemodynamic response function. For group analysis, a whole brain voxel‐wise three‐way repeated measures analysis of variance (RM‐ANOVA) was conducted for factors Phase (menstrual, follicular, ovulation, luteal), Stimulus (baseline, tingle, pain), and Task (simple motor, neutral, interference). Multiple comparisons correction was applied using minimum cluster size thresholds as determined by 3dClustSim.

Results for all statistical maps were displayed at a global *p* value of .001 corresponding to a minimum cluster size of 10 voxels in real space or 428.75 mm^3^. Post hoc contrast analyses were performed on peak coordinates in regions showing significant main or interaction effects and were considered significant if they resulted in a *p* value of .05 or below corrected for multiple comparisons with the adjusted false discovery rate (AFDR) (Benjamini & Hochberg, 2000).

### 2.6 Further statistical analysis

SPSS version 21 (IBM Corp., Armonk, NY, USA) was used to analyze MSIT performance metrics and psychophysical data considering a *p* value of .05.

## 3 RESULTS

### 3.1 Participant characteristics

Of the 15 participants completing the study, data of 12 right‐handed participants was analyzed (mean age: 28.9 ± 6.3 SD; range 22–40). Three participants were excluded from the analysis because blood hormone analysis revealed testing was done in the wrong cycle phase, excessive motion during the task paradigm in the MRI or because of equipment failure during the electrical stimulation in the MRI. Participant and menstrual cycle characteristics have been previously reported in detail (Meeker et al., 2020; Veldhuijzen et al., 2013).

### 3.2 Psychophysics

Electrical sensory detection thresholds demonstrated a trend for cyclic variation with less sensitivity during the menstrual compared to the follicular phase (*F* = 3.20, *p* = .051). Electrical pain thresholds did not significantly vary across the cycle (*F* = 1.05, *p* = .37). Also, neither pain intensity (*F* = 1.76, *p* = .19) nor unpleasantness (*F* = 1.17, *p* = .34), intended to be held constant by design, demonstrated significant cycle fluctuations during each session. Electrical stimulation intensity, in milliamperes, required to evoke moderate pain, remained stable across the menstrual cycle (*F* = 0.67, *p* = .44).

### 3.3 Multi‐Source Interference Task performance

**FIGURE 1:**
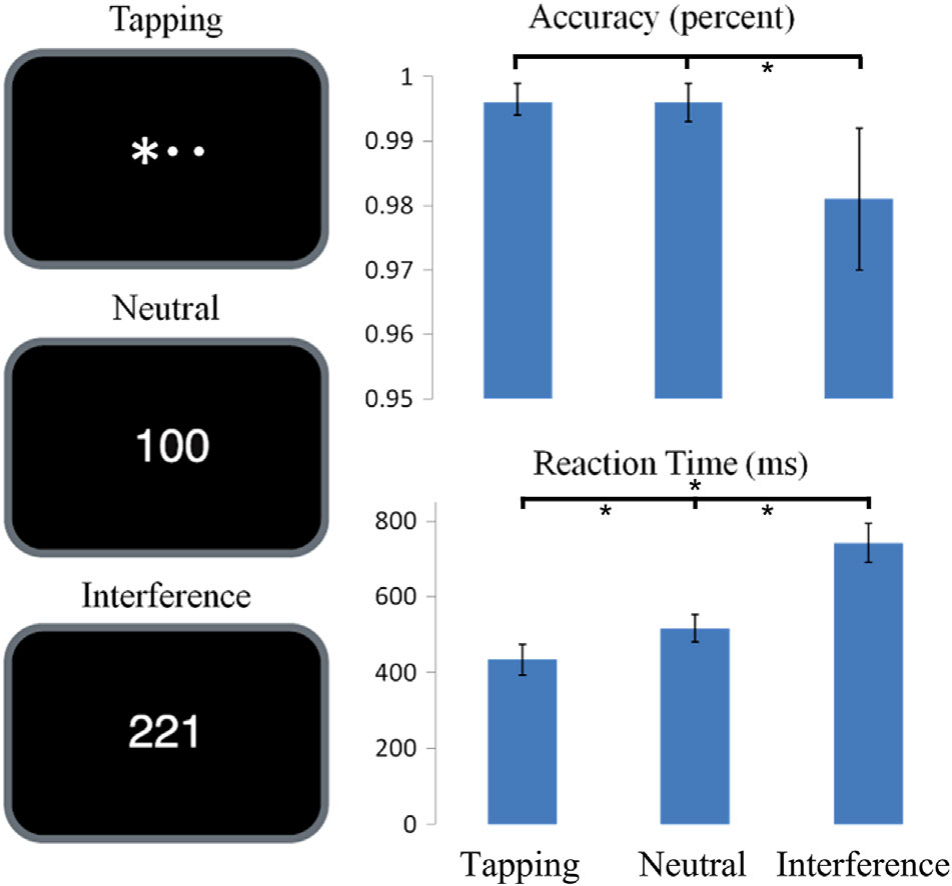
The Multi‐Source Interference Task. (a) The correct response is to push the first button in each condition in this example. (b) Subjects’ response on the interference trials was less accurate and reaction time significantly slower compared to the simple motor or neutral trials (mean ± 95% CI). *p < .05

Participants performed with a minimum accuracy of 85.5% on the interference task of the MSIT, 85.5% on the neutral and 94.0% on the tapping task. As expected, there was a significant effect of task difficulty on accuracy (*F* = 270.59, *p* < .001) and reaction time (7.71, *p* = .016; Figure 1). Cycle phase or electrical stimulus level did not affect accuracy levels or reaction times. None of the possible interaction effects were significant.

### 3.4 Brain activation patterns

**FIGURE 2:**
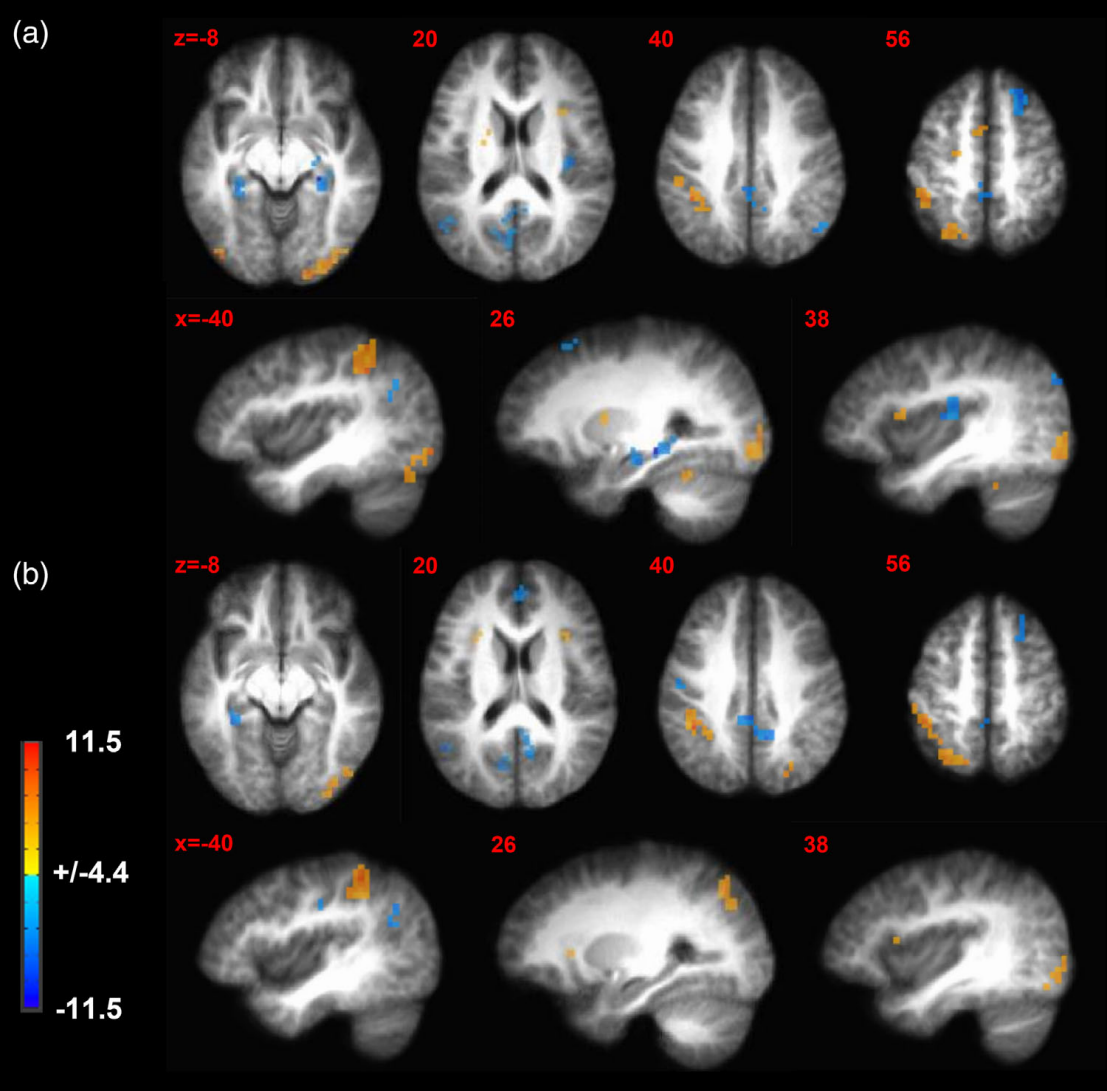
Brain activation patterns for task effects. (a) Contrast map where activations during the interference trials are significantly different (≥red and orange; ≤blue and light blue) compared to simple motor trial blocks. (b) Contrast map where activations during the interference trials are significantly different (≥red and orange; ≤blue and light blue) compared to neutral trial blocks. These contrasts are collapsed across menstrual cycle phase and stimulus intensity. *p* Value threshold = .001; minimum cluster size = 429 mm^3^

Brain activation patterns for *Task* are presented in Table 1 and Figure 2. In the contrasts between the interference and neutral tasks and between interference and simple motor tasks, we found the greatest activations in left inferior parietal lobule and bilateral inferior occipital lobule and greatest deactivations in bilateral precuneus, right anterior cingulate cortex (ACC), and left angular gyrus.

**TABLE 1:** Significant clusters from task main effect

						Significant contrasts					
Cluster location	BA	Side	Talairach (*x*, *y*, *z*)	Peak‐*F*	Volume (mm^3^)	I > N	I > SM	N > SM	N > I	SM > I	SM > N
Precuneus/paracentral lobule	5	L	(−2, −43, 48)	57.83	22209				^***^	^***^	^*^
Inferior parietal lobule	40	L	(−40, −43, 42)	63.91	13163	^***^	^***^	^**^			
Inferior occipital gyrus	18	L	(−40, −82, −8)	54.21	8318	^***^	^***^	^***^			
Angular gyrus	40	L	(−54, −64, 28)	39.33	6002				^***^	^***^	^**^
Medial frontal gyrus/supplementary motor area	6	L	(−5, 6, 52)	41.04	3602	^***^	^***^	^*^			
Dorsal precentral gyrus/middle frontal gyrus	6	L	(−23, −12, 59)	45.42	2916	^***^	^***^	^***^			
Superior frontal gyrus	8	L	(−19, 38, 45)	30.57	2873				^***^	^***^	
Parahippocampal gyrus	36	L	(−30, −36, −8)	32.14	2401				^***^	^***^	^*^
Cerebellar declive		L	(−2, −64, −11)	19.95	1929	^***^	^**^				
Precentral gyrus	4/6	L	(−44, −12, 34)	25.69	1801				^**^	^***^	
Putamen		L	(−23, −4, 14)	25.82	1758	^***^	^***^	^**^			
Premotor/middle frontal gyrus	6	L	(−47, 6, 42)	23.47	1629	^***^	^***^	^*^			
Hippocampus		L	(−26, −15, −14)	25.33	1501				^***^	^***^	
Dorsal anterior insula	13	L	(−26, 24, 17)	25.49	1415	^***^	^***^				
Anterior middle cingulate gyrus	32	L	(−9, 16, 42)	23.86	1115	^**^	^***^	^**^			
Caudate		L	(−9, 13, 10)	19.46	943					^***^	^***^
Inferior frontal gyrus pars opercularis	47	L	(−23, 34, 0)	22.99	943				^***^	^***^	^*^
Retrosplenial cortex	29	L	(−16, −40, 10)	23.72	943				^***^	^***^	
Cerebellar tonsil		L	(−2, −54, −32)	31.10	858	^***^	^***^				
Medial superior frontal gyrus	8	L	(−2, 38, 45)	13.55	772				^**^	^**^	
Cerebellar pyramis		L	(−5, −74, −28)	21.02	729	^**^	^***^	^**^			
Cerebellar tonsil		L	(−30, −46, −28)	18.02	643	^***^	^**^				
Dorsal posterior insula	13	L	(−33,−22, 20)	18.46	643				^***^	^***^	^*^
Inferior cerebellar semilunar lobule		L	(−33, −64, −39)	18.74	600				^***^	^***^	
Caudate		L	(−16, −22, 20)	38.72	557	^***^	^***^				
Anterior cingulate	32	R	(2, 44, 0)	29.27	9647				^***^	^***^	
Inferior occipital gyrus	17	R	(19, −92, −7)	69.60	7718	^*^	^***^	^***^			
Precuneus		R	(40, −71, 38)	29.58	3816				^***^	^***^	^**^
Dorsal anterior insula	13	R	(30, 24, 14)	32.96	3602	^***^	^***^				
Hippocampus		R	(26, −18, −14)	31.43	2958				^***^	^***^	
Superior frontal gyrus	6/8	R	(23, 24, 59)	36.86	2658				^***^	^***^	
Superior frontal gyrus	6	R	(26, −4, 66)	25.35	2572	^***^	^***^	^*^			
Culmen		R	(30, −46, −25)	41.52	2187	^***^	^***^	^**^			
Precentral gyrus	4	R	(58, −4, 31)	33.14	1629				^***^	^***^	
Superior parietal lobule	7	R	(23, −74, 52)	26.05	1544	^***^	^***^	^**^			
Putamen		R	(23, −1, 14)	30.84	1200	^***^	^***^	^*^			
Dorsal posterior insula	13	R	(37, −15, 20)	26.57	1072				^***^	^***^	
Cerebellar declive		R	(9, −74, −22)	38.88	1029	^***^	^***^	^*^			
Inferior parietal lobule	40	R	(33, −40, 45)	15.88	1029	^***^	^**^				
Superior parietal lobule	7	R	(30, −57, 45)	21.99	943	^***^	^***^				
Cerebellar declive		R	(33, −68, −22)	22.61	900	^***^	^***^	^*^			
Middle frontal gyrus/ premotor area	6	R	(47, 2, 38)	25.24	900	^***^	^***^	^*^			
Precuneus	7	R	(5, −74, 48)	16.95	858	^***^	^**^				
Inferior parietal lobule	40	R	(47, −29, 28)	19.56	815				^***^	^***^	
Postcentral gyrus	3	R	(37, −22, 45)	22.78	815				^**^	^***^	^***^
Cerebellar tonsil		R	(23, −50, −46)	16.65	772	^***^	^***^				
Middle temporal gyrus	21	R	(58, −40, 0)	19.51	472				^**^	^***^	^**^
Middle frontal gyrus	9	R	(30, 58, 20)	16.28	429				^***^	^***^	

^*^ *p* < .05.

^**^ *p* < .01.

^***^ *p* < .001.

Brain activation patterns for *Stimulus* are presented in Table 2. In the contrast between the pain and tingle levels, the greatest activations were found in bilateral posterior insula, right mid‐insula and putamen. The greatest deactivations were found in right pregenual ACC and left superior parietal lobule.

**TABLE 2:** Clusters from stimulus main effect

Region of interest	BA	Side	Talairach (*x*, *y*, *z*)	Peak‐*F*	Volume (mm^3^)	Significant contrasts P > T	P > B	T > B	T > P	B > P	B > T
Posterior insula	13	L	(−37, −22, 24)	29.13	986	^**^^*^	^***^				
Superior parietal lobule	7	L	(−33, −50, 56)	20.07	472				^**^	^***^	^*^
Posterior insula	13	R	(33, −22, 20)	97.25	4373	^***^	^***^	^***^			
MidInsula and putamen	13	R	(30, 10, 10)	26.88	1886	^***^	^***^				
Pregenual anterior cingulate	32	R	(2, 41, 3)	16.80	1029				^***^	^**^	
Frontal operculum	43/44	R	(54, −1, 14)	43.70	943	^***^	^***^				
Posterior insula	13	R	(40, −4, 0)	27.29	900	^***^	^***^				

^*^ = *p* < 0.05.

^**^ = *p* < 0.005.

^***^ = *p* < 0.001.

**FIGURE 3:**
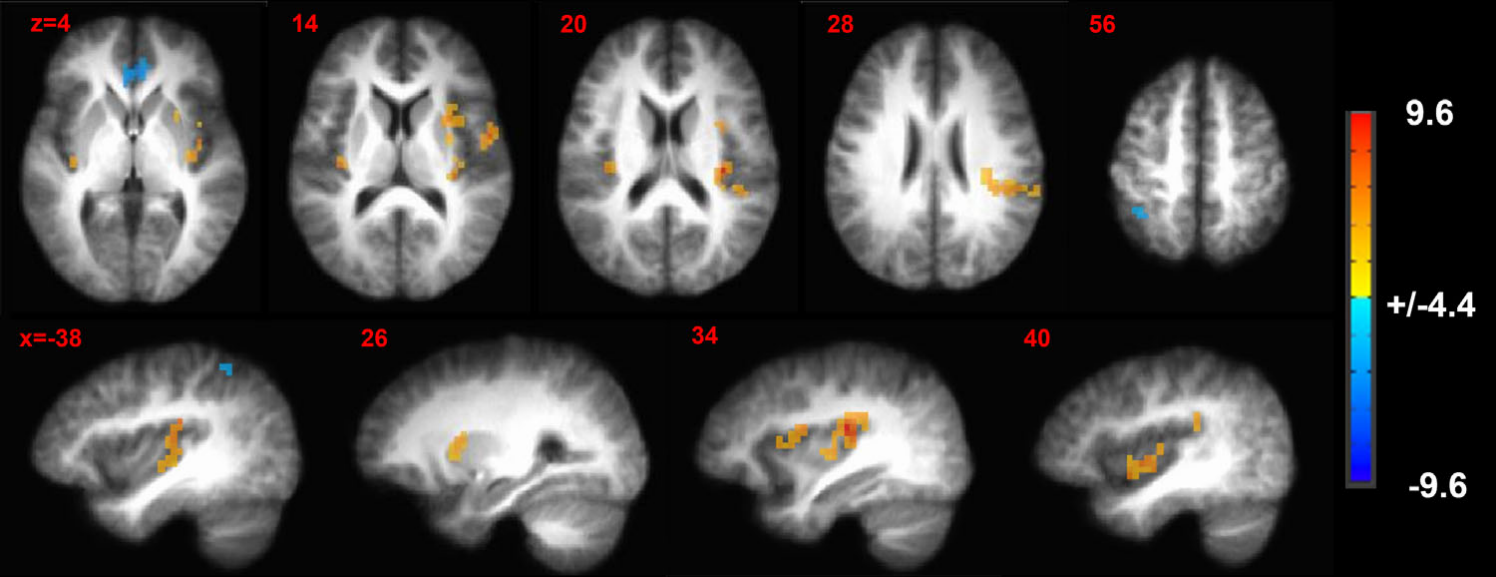
Brain activation patterns for sensory stimulation effects. Contrast map where activations during the painful stimulation trials are significantly different (≥red and orange; ≤blue and light blue) compared to electrical tingle trial blocks. These contrasts are collapsed across menstrual cycle phase and task difficulty. *p* Value threshold = .001; minimum cluster size = 429 mm^3^

**FIGURE 4:**
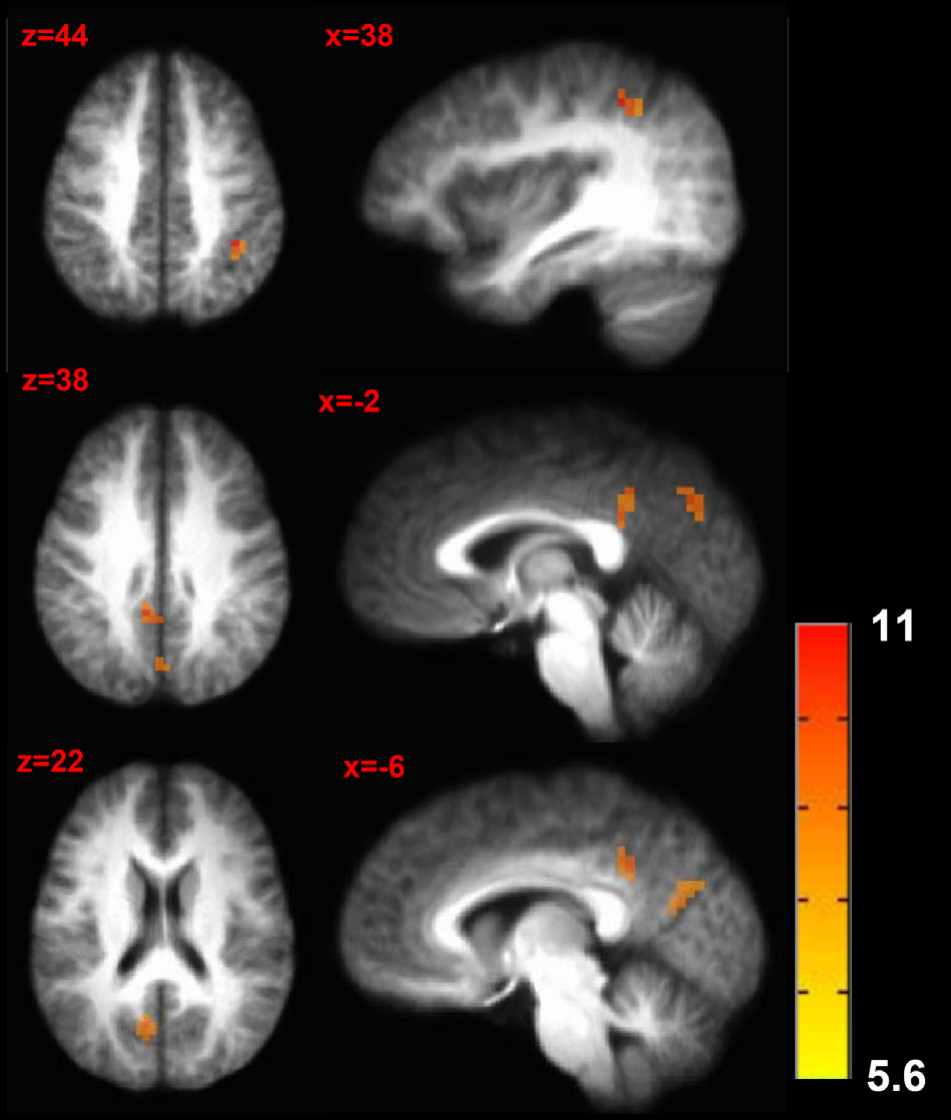
Brain activation patterns for the interaction between sensory stimulation and task effects. The *F*‐stat map of significant interaction effects between task difficulty and stimulus intensity is shown. *p* Value threshold = .001; minimum cluster size = 429 mm^3^

Sensory stimulation was found to interact with task performance in the left precuneus, left posterior cingulate cortex and right inferior parietal lobule (see Table 3; Figures 3 and 4).

**TABLE 3:** Cluster from stimulus by task interaction

Region of interest	BA	Side	Talairach (*x*, *y*, *z*)	Peak‐T	Volume (mm^3^)	Significant contrasts		*p* Value
Precuneus	7	L	(−2, −71, 34)	9.06	1286	None		1.99E‐05
Cingulate gyrus	23	L	(−9, −43, 38)	9.75	772	Task	SM > I	9.75E‐06
Inferior parietal lobule	40	R	(37, −43, 45)	10.98	472	Task	I > SM	2.92E‐06

No significant effects of *Phase* were found on brain activation patterns.

## 4 DISCUSSION

This study aimed to examine interactive brain activation patterns of pain and cognitive task performance. Additionally, the role of naturally fluctuating gonadal hormones on this relationship was explored. Significant interacting brain activation patterns for pain and cognition were found in regions representative of key regions involved in sharing of cognitive resources. These findings did not vary over the cycle.

Pain‐ and task‐related brain activation was found for several brain regions. In several instances, this (asymmetric) activation was due at least in part to participant responses being executed with the right hand during painful stimulation of the left foot. The interaction of brain activation during task performance and pain experience was found in the right (contralateral to pain stimulation) inferior parietal lobule, the left precuneus and the left cingulate gyrus. These regions have been previously reported to be involved in pain processing and the integration of different information sources. The inferior parietal lobule has been implicated in the sensory discrimination of pain, memory retrieval and expectancy violations (Bubic et al., 2009; Colloca et al., 2019; O'Connor et al., 2010; Oshiro et al., 2009). The precuneus has been related to diverse highly integrated functions including pain processing (Cavanna & Trimble, 2006; Zhang et al., 2020). The cingulate gyrus has been proposed to be involved in monitoring conflict, cognitive integration, and pain processing (Buckner et al., 2008; Greenspan et al., 2008; Jahn et al., 2016; van Veen & Carter, 2002; Vogt et al., 1996). The current study findings suggest that these areas are important integration regions for pain information and cognition processing. The specific laterality of these brain activation patterns needs further investigation, for instance, requiring a design where task and stimulation laterality is counterbalanced.

Previously, interacting effects of pain and cognition were reported in dorsolateral prefrontal, mid‐cingulate, and posterior parietal cortical regions, although other studies were also unable to demonstrate an interaction, or found conflicting results (Seminowicz & Davis, 2007a, 2007b; Seminowicz et al., 2004). These inconsistent findings in the neural representation of shared resources may be caused by variations in pain intensity levels or cognitive load in the examined paradigms: high pain may interfere with cognitive task performance more, while higher cognitive load may produce enhanced distraction from pain (Eccleston, 1994; Meeker et al., 2021; Moore et al., 2017; Veldhuijzen et al., 2006). The MSIT task was specifically chosen in the current study as it has been shown to reliably elicit brain activations in the cingulo‐frontal‐parietal attention network reflecting cognitive processing (Bush & Shin, 2006). Nevertheless, the specific involvement of brain areas under different cognitive task and pain conditions needs to be examined further in future studies. Further studies into this field seem to be indicated given that cognitive impairments on a variety of tasks have been demonstrated in pain patients, while functional and structural brain alternations potentially underlying these behavioral assessments have been shown. The importance of our enhanced understanding of these interactions is illustrated by brain imaging findings showing that cognitive strategies can successfully modulate pain activations in the brain and as such serve as a potential therapeutic target (Seminowicz et al., 2013).

In addition, this study showed that gonadal hormones did not clearly interact with task or pain processing. This is contrary to our previous report showing that fluctuating gonadal hormones interacted with pain processing (Veldhuijzen et al., 2013). A potential explanation of these conflicting findings could be that we used a different pain modality in this study compared to the previous study. Previously, pressure pain instead of electrical pain was used. It has been previously shown that electrical pain stimuli produce a different response profile over the cycle than other pain types, possibly explaining these differential effects (Riley, Robinson et al., 1999). It is therefore recommended for future studies to examine the role of gonadal hormone fluctuations in a design that has been shown to be sensitive to these changes (e.g., making use of pressure pain stimulations). Another possible explanation could be the relatively low sample size of the current study, although this sample size was directly comparable to our previous report that demonstrated significant findings using a similar within‐subjects design (Veldhuijzen et al., 2013). Since sex differences have been reported in the cognitive processing of pain, more studies are needed in this understudied research field (Romano et al., 2019).

Strengths of this study were that we used validated procedures including electrical pain applications, which were combined with a previously validated cognitive task performance measure for brain imaging purposes. Moreover, we used a within‐subjects design of four sessions over different menstrual cycle phases with start sessions randomized per phase, which allowed us to control for order effects. Also, hormone assays in blood confirmed correct cycle phase assessments. The main limitation of the study was the relatively low sample size despite the within‐subjects design that was chosen.

In sum, the study findings demonstrate the neural correlates of shared resources between pain and cognition and as such provide direct support of the limited resource theory at the neurofunctional level in humans. The influence of fluctuating gonadal hormones on the interaction between pain and cognition was not demonstrated but this finding needs to be repeated in larger samples. These findings are of relevance as they provide further insights into the potential mechanisms behind cognitive performance difficulties in pain patients and may facilitate the development of novel pain‐relieving treatment strategies focusing on specific cognitive factors such as cognitive interference.

## FUNDING

This work was supported by P50‐AR049555 (JDG), the University of Maryland General Clinical Research Center (GCRC) NIH NCRR Grant M01‐RR16500, and the University of Maryland, Baltimore Center to Advance Chronic Pain Research.

## CONFLICT OF INTEREST

The authors declare no conflicts of interest.

## AUTHOR CONTRIBUTIONS

DSV: Design, data collection, data interpretation, draft and revise manuscript, final approval. TJM: Analyze data, data interpretation, preparing figures, draft and revise manuscript, final approval. DT: Data collection, data interpretation, draft and revise manuscript, final approval. MLK: Data collection, analyze data, data interpretation, preparing figures, revise manuscript, final approval. RPG: Design, data interpretation, final approval. JDG*: Design, data interpretation, revise manuscript. *The senior author passed away before final submission of the article but contributed significantly up until the prefinal version and approved all analyses and result reporting.

### PEER REVIEW

The peer review history for this article is available at https://publons.com/publon/10.1002/brb3.2593.

## Data Availability

The data that support the findings of this study are available on request from the corresponding author. The data are not publicly available due to privacy or ethical restrictions.
